# Diet and Hunting Range of Wintering Long-Eared Owls (*Asio otus*) Depend on Land Use

**DOI:** 10.3390/biology15030269

**Published:** 2026-02-02

**Authors:** Dávid Szép, Jenő J. Purger

**Affiliations:** 1 Department of Ecology, Institute of Biology, University of Pécs, Ifjúság útja 6, 7624 Pécs, Hungary; szep.david@pte.hu; 2 Institute of Physiology, Medical School, University of Pécs, Szigeti út 12, 7624 Pécs, Hungary; 3 BioRes Limited Partnership, Barackvirág utca 27, 7624 Pécs, Hungary

**Keywords:** *Asio otus*, habitat preference, landscape structure, pellet analysis, small mammals, Hungary

## Abstract

Long-eared Owls (*Asio otus*) overwinter in groups, mainly on coniferous trees in human settlements. These owls regurgitate the indigestible parts of their prey as pellets. Analysis of pellet contents provides valuable information on diet composition and reflects the characteristics of the hunting area. We assumed that prey availability for owls is influenced by the extent of built-up areas and by the distribution of habitats around the roosting site. In the winter of 2016/2017, we collected three samples of 1000 pellets from settlements in the southern part of Hungary: the village of Udvar, the town of Mohács, and the city of Pécs. Species accumulation curves were calculated based on cumulative prey species richness and rarity. To assess the effect of built-up areas, we compared the diet composition of owls wintering in settlements of different sizes. To estimate the size of the hunting area, we compared the relative abundance of small mammal species with different habitat preferences detected in the pellets with the proportion of habitat type within circles of one, two, and three kilometres around the roosting sites. The proportion of open and forested habitats decreased as the extent of built-up areas increased. This reduced access to the owls’ main prey, especially Common Voles (*Microtus arvalis*) and other small mammals. As a result, the minimum number of pellets needed for a representative sample was at least 300 in the village, 600 in the town, and 1000 in the city. In the city, owls showed higher prey species richness, greater diversity, higher evenness, and a wider food niche than owls in the town or village. In the village, the relative abundance of small mammal species associated with urban, open, forested, or wetland habitats in the owl diet fully matched the distribution of these habitat types within a three-kilometre radius around the roosting site. In the town, such matching was found only for open and forested habitats. In the city, this relationship was observed only for wetland habitats. Pellet analysis of wintering Long-eared Owls can reveal the impact of land use on small mammal prey communities and highlight important ecosystem services of this owl species, including its role in reducing small mammal pests.

## Simple Summary

Long-eared Owls often roost in coniferous trees during winter within human settlements. These owls regurgitate pellets with the remains of their prey. Analysis of the content of these pellets can show what prey is available in the surrounding landscape. We studied whether the amount of built-up land around owl roosts affects the availability of prey for owls. During one winter, we collected large numbers of pellets from a village, a town, and a city in southern Hungary. As built areas increased, open fields and forests decreased, limiting access to the owls’ main prey, especially voles and other small mammals. In more urban places, owls ate a wider variety of prey, but each type made up a smaller share of the diet. The match between prey in the diet and nearby habitats was strongest in the village and weakest in the city. Our results show that owl pellets can help track how land-use changes affect small mammal communities, and they highlight the owl’s valuable role in naturally controlling pest species.

## 1. Introduction

The Long-eared Owl (*Asio otus*) is a medium-sized owl with a Holarctic distribution [1]. From late autumn to early spring, it usually winters in groups on coniferous trees within human settlements [2,3,4,5,6,7]. Depending on the number of owls present, a large number of pellets accumulate under these trees. These pellets consist of indigestible remains of small mammal and bird prey [8,9,10,11,12,13]. Although the diet of the Long-eared Owl is less diverse than that of many other owl species, analysis of a sufficiently large number of pellets provides detailed information on the small mammal community within its hunting range. This method can be more informative than conventional trapping [1,14]. To accurately characterize the small mammal community of an area, a representative pellet sample is required. The size of such a sample may depend on habitat conditions and land use around the roosting site. The primary prey of the Long-eared Owl is the Common Vole (*Microtus arvalis*), which is a serious agricultural pest [10,15]. Therefore, the owl plays an important role in regulating the vole population [16]. When Common Voles are scarce within the hunting area, the proportion of mice or birds in the owl’s diet increases [17,18]. Owls wintering in cities often face limited prey availability. As a result, they may hunt at greater distances from their roosts [19] or switch to alternative prey species [18,20,21,22].

The distribution and structure of habitats within the hunting range influence prey availability and accessibility. These factors are reflected in the diet composition of Long-eared Owls and also affect the size of their home range [9,19]. Radio telemetry studies have shown that home range size depends on the sex of the birds, as females show reduced movement frequency and restrict their home range to considerably smaller areas during incubation and throughout the first weeks post-hatching [23], and their activity was studied during the breeding season [19,21,23,24] or during winter [2,25,26]. The size of the home range is generally larger in winter due to the lower availability of prey species and the high number of owls roosting together [25]. The degree of urbanization and extent of built-up areas around roosting sites also influence how far owls must travel to hunt [19,24]. Several studies suggest that Long-eared Owls may hunt at distances of three kilometres or more from their roosts [2,23,24,25,26]. Individually marked owls often leave the roost in different directions on the same night, which results in partially separated hunting areas [26]. For individual owls, these areas can be represented by relatively small circles around the roost. In contrast, owls wintering in large groups may collectively cover much larger areas. Due to spatial separation among individuals, these areas may extend to a radius of 3 km or more [25]. However, most published estimates of home range size do not exceed a 2 km radius [9,19,27,28], even though owls may hunt over larger areas.

The foraging behaviour of the Long-eared Owl is influenced by several factors, including weather conditions, prey size, prey accessibility, and the proportion of habitat types within the hunting range [9,10,11,13,22,29,30]. Studies that directly examine the relationship between habitat structure and the relative abundance of small mammal prey in the diet of this species are limited [9,14,31]. Previous research has analyzed landscape structure within hunting areas defined by circles with radii of 0.5, 0.7, 1, 1.13, 3, and 5.6 km [9,22,27].

The aim of this study was to determine the following: (1) the pellet sample size required to reliably represent the prey composition of Long-eared Owls; (2) whether the diet composition differs among owls wintering in settlements of different size; and (3) whether the distribution of small mammals with different habitat preferences detected in pellets overlaps with the distribution of their habitats, and whether this overlap can be used to estimate the size of the hunting area.

## 2. Materials and Methods

### 2.1. Study Area

**Figure 1:**
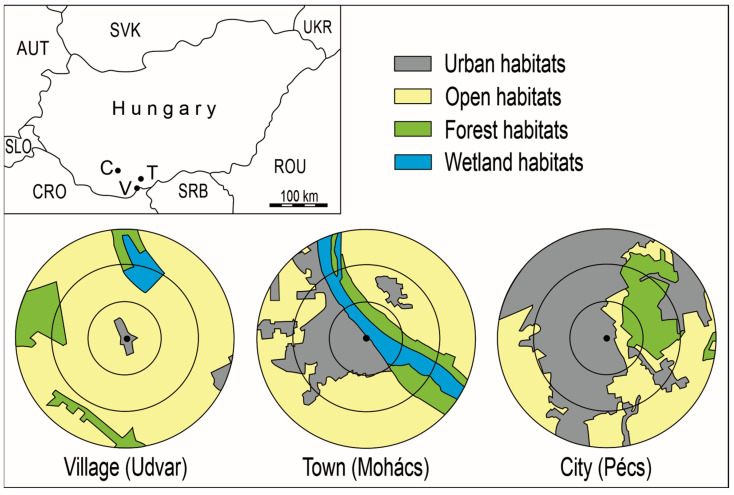
The location of the Long-eared Owl roosting sites (V—village, T—town, and C—city), and the distribution of the habitat types in the circle with a radius of one, two, and three kilometres.

Pellet collection was conducted between December 2016 and March 2017 in three settlements of different sizes and degrees of urbanization in the South Transdanubia region of Hungary. Udvar is a small village with 122 inhabitants, located near the Hungarian–Croatian border and surrounded by agricultural land (Figure 1). Approximately 20 Long-eared Owls wintered on Norway Spruce (*Picea abies* (L.) H. Karst.) trees in a yard in the village centre. The town of Mohács lies about 10 km north of Udvar, on the right bank of the Danube River, and it has 17,315 inhabitants. Apart from riparian forests along the river, the surrounding landscape is dominated by agricultural fields (Figure 1). In streets close to the river, European Nettle Trees (*Celtis australis* L.) and Silver Birches (*Betula pendula* Roth) served as roosting sites for approximately 70 owls. The city of Pécs is located 37 km west of Udvar and 36 km west of Mohács, on the southern slopes of the Mecsek Mountains. In the southern part of the city, around 40 owls regularly roosted in Norway Spruce trees in a public cemetery. This roosting site is mainly surrounded by an urban environment; however, gardens, arable land, grasslands, and forests are present to the east (Figure 1). The climate of the study area is moderately warm and moderately dry, with an average annual temperature of 10.0–10.8 °C and annual precipitation of 600–670 mm [32].

### 2.2. Collection and Analysis of Pellets

Based on previous studies, a sample of 1000 pellets is considered sufficient to represent the diet composition of Long-eared Owls [22]. Therefore, all pellets found under the roost trees were collected at each site on six occasions on the same day. From the total material, intact pellets were randomly selected for each month in proportion to the number of pellets collected (December: 300; January: 400; February: 200; and March: 100), resulting in 1000 pellets per site. Each pellet was individually wrapped to prevent damage and mixing of contents. Pellets were then grouped into three sets of ten bags, each containing 100 pellets, and processed in random order. Dry pellets were dissected using tweezers, and bone remains were cleaned with toothbrushes [22]. Small mammal and bird species were identified based on osteological characteristics using published identification keys [33,34,35,36,37,38,39]. The number of prey individuals was determined by counting skulls and corresponding mandibles.

### 2.3. Landscape Structure and Statistical Analysis

Species accumulation curves were generated for each site using taxon counts from the 100-pellet bags to characterize how the cumulative number of detected species increases with the increasing number of pellets. To estimate the minimum representative sample size, individual-based rarefaction curves were calculated from abundance data, expressing the expected number of taxa in smaller samples using PAST software Version 5.3 [40].

For diversity analysis, small mammal species and bird genera were considered (damaged Wood mouse (*Apodemus* sp.) remains and unidentified bird (Aves) remains were excluded). Prey diversity was quantified using the Shannon diversity index (H) and Pielou’s evenness index (J) [40]. Food niche breadth was calculated from relative prey abundances using the Simpson index [41]. Niche overlap between sites was assessed using the Renkonen index (Pjk) [42]. Differences in relative prey abundances, diversity indices, and niche breadth among sites were tested using the Mann–Whitney U test [40]. Only statistically significant differences (*p* < 0.05) are presented in the results.

Previous studies indicate that Long-eared Owls typically capture most of their prey within a distance of up to 3 km from their breeding or roosting sites [2,19,23,24,25]. Therefore, circular buffers with radii of 1, 2, and 3 km were defined around each roosting site. Habitat composition within these circles was analyzed using CORINE Land Cover 2018 maps [43]. CORINE land-cover categories were grouped into four habitat types: urban, open, forest, and wetland (Figure 1). The proportional area of each habitat type was calculated for each radius around the roosting sites (Table 1).

Small mammal species detected in the pellets were assigned to four types according to their primary preferred habitat, as given in an atlas of mammals in Hungary [44]. These groups corresponded to the habitat categories used in the landscape analysis. The relative abundance of these prey groups in owl diet was compared with the proportional availability of the corresponding habitat types within the assumed hunting areas using the G-test for homogeneity [45]. Differences among sites were tested using the Mann–Whitney U test [40].

## 3. Results

### 3.1. Size of a Representative Pellet Sample

**Figure 2:**
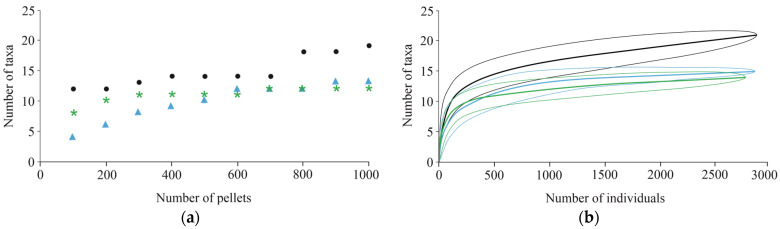
Variation in the number of taxa detected from randomly collected sets of 100 pellet samples across the three sampling sites (village—green, town—blue, and city—black): (**a**) cumulative taxa number; and (**b**) individual rarefaction with increasing sample size (standard errors converted to 95% confidence intervals).

Comparison of cumulative taxon numbers from 100-pellet batches collected at the three sites showed clear differences among settlement types. In the village (Udvar), the number of detected taxa increased only slightly after analyzing 300 pellets. In the town (Mohács), this threshold was reached at approximately 600 pellets. In contrast, in the city (Pécs), the species accumulation curve did not reach a clear plateau, even at 800–1000 pellets, indicating that a relatively large sample is required to capture maximum taxon richness (Figure 2a).

Individual-based rarefaction analysis, calculated from the number of prey individuals, showed that the curve approached a plateau at around 1000 individuals. In the city of Pécs, where prey composition was more diverse, taxon richness continued to increase beyond 1000 individuals (Figure 2b). When only small mammal prey was considered, analysis of 500 pellets per site was sufficient to detect all potential small mammal species, regardless of settlement type.

### 3.2. Relationships Between Owl Prey Composition and Settlement Size

During the winter of 2016/2017, small mammals strongly dominated the diet of Long-eared Owls at all three sites (village: 99.86%, town: 99.51%, and city: 97.79%). Only occasional bird remains, and a single insect fragment were found in the city (Pécs) sample. The most frequent prey species at all sites was the Common Vole, followed by the Wood Mouse (*Apodemus sylvaticus*). The third most frequent species was the Steppe Mouse (*Mus spicilegus*) in the village (Udvar) and the city (Pécs), while in the town (Mohács) it was the Striped Field Mouse (*Apodemus agrarius*) (Table 2). The mean number of prey items per pellet was 1.39 in the village, 1.44 in the town, and 1.45 in the city.

The number of detected prey taxa was lower in the village of Udvar (12) and in the town of Mohács (13) than in the city of Pécs (19). As a result, Shannon diversity, evenness, and food niche breadth were significantly higher in the city than in either the town or the village (Table 3). These values did not differ significantly between the village and the town. Dietary niche overlap was highest between the village and the town (89.22%), followed by overlap between the village and the city (70.34%). The lowest niche overlap was observed between the town and the city (66.70%).

**Figure 3:**
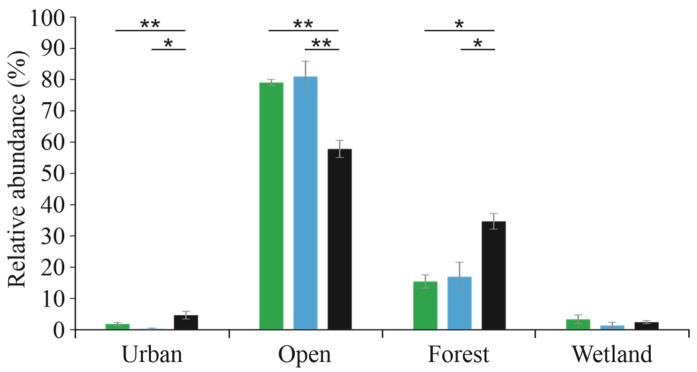
Relative abundance (±SE) of mammals with different habitat preferences in the diet of Long-eared Owls across the three settlements (village—green, town—blue, and city—black) and their comparison (* *p* < 0.05, ** *p* < 0.01).

Owls, wintering in the city (Pécs), had significantly higher proportions of small mammal species associated with urban and forest habitats than owls in the village (Udvar) and the town (Mohács). In contrast, species preferring open habitats were significantly more frequent in the village and the town than in the city (Figure 3). No significant differences were detected among settlements for wetland-associated species. There were no significant differences between the village and the town for any habitat category.

**Figure 4:**
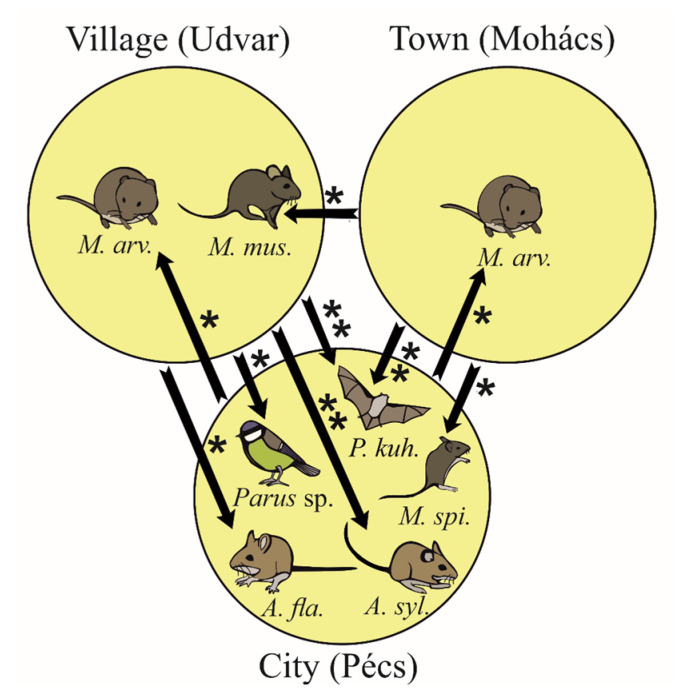
The significant differences (* *p* < 0.05, ** *p* < 0.01) in the relative abundance of the prey taxa between the three different sampling sites (arrows point to the significantly higher value) (*A. fla.*—*Apodemus flavicollis*, *A. syl.*—*Apodemus sylvaticus*, *M. arv*—*Microtus arvalis*, *M. mus.*—*Mus musculus*, *M. spi.*—*Mus spicilegus*, and *P. kuh.*—*Pipistrellus kuhlii*).

Significant differences in relative abundance were detected for six mammal species and one bird genus (Figure 4). Kuhl’s Pipistrelle (*Pipistrellus kuhlii*) occurred only in the city of Pécs, resulting in significantly higher relative abundance there compared to the village (*Z* = −3.17, *p* = 0.001) and the town (*Z* = −2.96, *p* = 0.002). The Common Vole was significantly more frequent in the village (*Z* = 2.43, *p* = 0.014) and the town (*Z* = 2.36, *p* = 0.018) than in the city. The Wood Mouse (*Z* = −2.57, *p* = 0.008) was significantly more frequent in the city than in the village, and the Yellow-necked Mouse (*Apodemus flavicollis*) showed a similar pattern (*Z* = −2.45, *p* = 0.014). The Eastern House Mouse (*Mus musculus*) was more frequent in the village (*Z* = 2.39, *p* = 0.017), while the Steppe Mouse was more frequent in the city (*Z* = −2.11, *p* = 0.030). Tits (*Parus* sp.) were also significantly more frequent in the city samples than in the village samples (*Z* = −2.50, *p* = 0.014).

### 3.3. Relationship Between Prey Habitat Preferences and Habitat Composition of Hunting Areas

Comparisons between the relative abundance of prey with different habitat preferences and the proportion of corresponding habitat types within circles of 1, 2, and 3 km radii revealed several cases where no significant differences were detected (Table 4). Within a 1 km radius, open habitats around the village (Udvar), forest habitats around the town (Mohács), and wetland habitats around the city (Pécs) showed similar proportions to those of their associated prey species. At a radius of 2 km, the same pattern was observed for the town and the city, while in the village, only forest-associated species differed significantly from habitat proportions. Within a 3 km radius, the relative abundance of all four prey groups with different habitat preferences matched the proportions of their corresponding habitat types around the village. In the town, this matching was observed only for species associated with open habitats. In the city, where urbanization was extensive, only wetland-associated species showed a similar pattern, while urban-, open-, and forest-associated species differed significantly from the proportions of their corresponding habitats.

## 4. Discussion

Our results showed that the number of pellets required for a representative diet sample strongly depends on the degree of urbanization around the Long-eared Owl roost. In the village and small town, where owls can hunt in extensive open agricultural areas close to the settlement, 300–600 pellets were sufficient to detect most small mammal species. In contrast, near the edge of a larger city, where open habitats were scarce, the proportion of Common Voles in the diet was lower, and a wider range of alternative prey species occurred. After rapid saturation of common species, rare taxa continued to appear, resulting in a longer plateau phase in the species accumulation curve compared to less urbanized sites. The point at which the curve stabilized was therefore strongly influenced by landscape structure and land use. In the village dominated by open agricultural habitats, Common Voles were abundant, so fewer pellets were needed to characterize the diet. In urban environments where prey diversity was higher, new taxa continued to appear, even after analyzing 800 pellets. Collecting large numbers of pellets from communal roosts is usually easy, and their analysis provides a reliable picture of diet composition [8,9,10,11,12,13].

In all three settlements, small mammals made up more than 97% of the winter diet of Long-eared Owls, which is slightly higher than values reported for Central Europe [10]. The most frequent prey species was the Common Vole, which is the primary prey of this owl in Central Europe [46,47,48,49]. Vole availability strongly affects owl behaviour and survival [4,9]. When voles are scarce, owls switch to alternative prey [10,46,50,51], which increases diet diversity [9,52]. Our results support this pattern. The sample from the city of Pécs contained significantly more prey taxa than samples from the village and the town. When the main prey is limited, Long-eared Owls capture less frequent species [13,46,53]. As a result, vole abundance is negatively correlated with prey species richness [4].

Due to lower vole abundance and higher prey diversity, owls wintering in the city showed higher diet diversity, evenness, and niche breadth. When the main prey population declines, owls broaden their realized niche to reduce competition [54]. Common Vole dominance is negatively related to niche breadth [4,30]. In urban habitats, diet breadth increases because mice and rats, such as the Wood Mouse, Yellow-necked Mouse, and Brown Rat (*Rattus norvegicus*), become more available [11].

Changes in settlement size and land use alter habitat availability for small mammals, which is reflected in the diet of this prey-specialist owl [9,52]. From village to town to city, built-up areas increase, while open habitats decrease, and this pattern is clearly reflected in prey composition. No major differences were found between the village and the town, as both had extensive open habitats within 3 km of the roost [1,55]. In contrast, in the city, species preferring open habitats were much less frequent, which is consistent with their limited availability. Species associated with forest and urban habitats were more common, reflecting their higher coverage within the assumed hunting area. Reduced availability of open habitats and Common Voles forced Long-eared Owls to rely more on alternative prey species associated with forest and urban habitats [9,52].

The lowest relative abundance of Common Voles was observed in the city of Pécs. This is likely caused by the limited availability of open (agricultural) habitats near the roosting site, which are preferred by this species [56]. In contrast, the Wood Mouse, Yellow-necked Mouse, and Brown Rat were more frequent in the city samples. In Central Europe, Wood Mice are the most common substitute prey for voles in the diets of Long-eared Owls [4,10]. Urban pellet samples often contain rat remains when vole abundance is low [13]. The presence of Kuhl’s Pipistrelle in pellets from the city of Pécs also indicates vole scarcity around the roost. When owls cannot capture enough small mammals, bats may become an important alternative prey [4,57]. Although most Hungarian bats hibernate in winter, acoustic studies with detectors show that some species, including Noctule (*Nyctalus noctule*), Savi’s Pipistrelle (*Hypsugo savii*), and Kuhl’s Pipistrelle (detected in our study), fly on mild winter days in urban areas [58]. These latter two species are currently expanding from the Mediterranean region and often roost in buildings [58]. The Steppe Mouse was also more frequent in the city than in the town. Similar patterns have been observed in urban areas of Bulgaria, where this species represents an important alternative prey for Long-eared Owls [59]. Between the two smaller settlements, the only clear difference was the higher proportion of Eastern House Mouse in the village. A small village with backyard livestock farming, stables, barns, and feed storage areas provides suitable winter shelters for the Eastern House Mouse [60]. In some cases, the Eastern House Mouse can even dominate in the winter diet of Long-eared Owls [17,18]. Birds were also more frequent in the city sample, again reflecting vole scarcity [13,61]. Owls typically hunt small birds such as tits and sparrows along forest edges and in parks [4,53].

Comparisons between small mammals with different habitat preferences and habitat proportions within 1, 2, and 3 km radii of the presumed hunting area showed that built-up areas around roosts exceeded the proportion of urban-preferring prey species in the diet, even within a 1 km circle. In larger settlements, urban habitat remained dominant, even at greater distances, but urban-preferring species were under-represented in the diet. This supports earlier findings that Long-eared Owls avoid built-up areas during hunting due to low prey availability or poor accessibility [19,24,62]. As urbanization increases, owls expand their hunting range [24] and occur less frequently in landscapes dominated by artificial surfaces [26].

Species preferring open habitats were abundant in pellets collected in the village, closely matching the high availability of open habitats around the roost. In the town, Owls likely had to travel farther to reach suitable hunting areas. In the city, open habitats were fragmented or distant, suggesting elongated and irregular hunting ranges [11]. In addition to prey size specialization, Long-eared Owls also show clear habitat selection [30], preferring open areas [1,55] because their main prey, the Common Vole, is strongly associated with arable land and agricultural crops [10,63].

Forest habitats covered relatively small areas around the village and the city, though the relative abundance of the forest-preferring species exceeded the mapped forest area. Around the town, riparian forests along the Danube River provided an extensive forest habitat, and their proportion did not differ significantly from the relative abundance of forest-associated prey species. Long-eared Owls likely capture forest species along forest edges, parks, tree lines, and riparian corridors [19,24,64]. Many of these linear habitat elements were not shown on Land-cover maps due to their small size or classification within other categories (such as land principally occupied by agriculture, with significant areas of natural vegetation). Similarly, Owls can also prey on small mammals that prefer forest habitats in the shrubbery and tree rows at the edges of small wetlands, which are also missing from the map [26].

In the city, wetlands were small and consisted mainly of channelized streams lacking typical riparian vegetation. In the town, wetland-preferring species were absent because riparian zones were dominated by forests or built-up areas rather than reedbeds or sedge stands preferred by these species [1,55]. Wetland type strongly influences small mammal communities, and differences are pronounced, especially when adjacent habitats vary (e.g., streams, marshes, and rivers) [65]. Owls likely capture wetland species along riparian edges [19,24,64], but these narrow linear habitats were not represented in the maps.

Overall, our results suggest that a 3 km radius best represents the hunting areas of wintering Long-eared Owls in landscape ecological studies, as this scale most accurately reflects prey habitat preferences. Our findings also confirm that the degree of urbanization strongly influences hunting range shape and size [19,24]. Although previous studies often used radii of 1 km or less [9,19,27], our results indicate that even small settlements can have relatively high proportions of built-up areas that may bias conclusions. In the city, no clear trend emerged with increasing radius, suggesting an irregular hunting range focused on open habitats outside the urban core [11].

Pellet analysis provides valuable indirect information on small mammal communities and their relative abundance within potential hunting areas [66]. If pellet collection is carried out over multiple years, the effects of weather fluctuations and variations in prey population numbers, such as vole population cycles, can be reduced or avoided [22]. Although Barn Owl (*Tyto alba*) pellets are far more suitable for this purpose, partly due to the species’ opportunistic foraging behaviour [67]. This method has several limiting factors, such as the resolution of the maps mentioned earlier and the fact that the prey species is not restricted to a single habitat type. In addition, Long-eared Owls also show prey preferences, which means that the results obtained should be interpreted with appropriate caution. Despite this, analysis of large pellet samples from wintering Long-eared Owls can therefore reveal the effects of land use on small mammal prey communities [68,69] and highlight the important ecosystem services provided by this owl species, including its role in reducing small mammal pests [70].

## 5. Conclusions

Our results clearly highlighted the importance of determining a representative pellet sample size, as it strongly depends on the degree of urbanization and land use around the owl roosting sites. To describe the diet composition of Long-eared Owls wintering in the village or small town surrounded by agricultural land, a sample of at least 500 pellets is needed, while for those wintering in cities, analyzing at least 1000 pellets is recommended. The prey composition of owls wintering in larger cities is more diverse, and in addition to small mammals, certain taxa in the prey become more abundant. If the assumed hunting area is approximately a circle with a 3 km radius around the roosting site, where urbanization is low, then the habitat preference of small mammal species detected in pellets overlaps with the distribution of habitats in this area. In such cases, pellet analysis can be used for monitoring, to reveal changes in small mammal communities, and to indirectly conclude on changes in land use within the owl’s hunting range.

## Figures and Tables

**Table 1:** The proportion of habitats (%) around Long-eared Owls’ roosting sites in the circle with a radius of 1, 2, and 3 km.

	Settlement Type	Village (Udvar)	Town (Mohács)	City (Pécs)
1 km	Urban habitats	8.1	53.9	78.2
	Open habitats	91.9	5.6	12.7
	Forest habitats	0.0	14.6	9.0
	Wetlands	0.0	26.0	0.0
2 km	Urban habitats	2.1	35.3	63.9
	Open habitats	92.9	38.1	17.1
	Forest habitats	1.9	13.0	19.0
	Wetlands	3.1	13.6	0.0
3 km	Urban habitats	2.2	20.4	53.3
	Open habitats	85.3	60.9	34.6
	Forest habitats	9.8	9.6	12.1
	Wetlands	2.7	9.1	0.0

**Table 2:** Number of specimens (n_i_) and relative abundance of prey taxa (%) in the three different sampling sites, and habitat preference (hp) of prey: u, urban; o, open; f, forest; and w, wetland habitats.

Settlement Type		Village (Udvar)		Town (Mohács)		City (Pécs)	
Prey	hp	n_i_	%	n_i_	%	n_i_	%
*Crocidura suaveolens*	o	1	0.07	4	0.28	0	0.00
*Pipistrellus kuhlii*	u	0	0.00	0	0.00	36	2.48
*Microtus lavernedii*	w	4	0.29	2	0.14	1	0.07
*Microtus arvalis*	o	1025	73.32	1125	77.96	698	48.10
*Microtus subterraneus*	f	8	0.57	5	0.36	4	0.28
*Arvicola amphibius*	w	0	0.00	0	0.00	1	0.07
*Myodes glareolus*	f	2	0.15	4	0.28	3	0.21
*Apodemus agrarius*	f	66	4.72	47	3.26	81	5.58
*Apodemus flavicollis*	f	15	1.07	14	0.97	58	4.00
*Apodemus sylvaticus*	f	98	7.01	137	9.50	303	20.88
*Apodemus* sp.		35	2.50	42	2.91	49	3.38
*Micromys minutus*	w	44	3.15	18	1.25	33	2.27
*Mus musculus*	u	26	1.86	6	0.41	21	1.45
*Mus spicilegus*	o	72	5.15	32	2.22	121	8.34
*Rattus norvegicus*	u	0	0.00	0	0.00	10	0.69
Aves (*Aegithalos* sp.)		0	0.00	0	0.00	1	0.07
Aves (*Carduelis* sp.)		0	0.00	1	0.07	1	0.07
Aves (*Fringilla* sp.)		0	0.00	0	0.00	1	0.07
Aves (*Passer* sp.)		0	0.00	0	0.00	7	0.48
Aves (*Parus* sp.)		1	0.07	5	0.36	13	0.89
Aves		1	0.07	1	0.07	8	0.55
Insecta (Hymenoptera)		0	0.00	0	0.00	1	0.07
Total (∑)		1398	100.00	1443	100.00	1451	100.00

**Table 3:** Mean diversity indices of Long-eared Owl prey species and pairwise differences among settlements, assessed using the Mann–Whitney U test.

	Settlement Type		Village (Udvar)			Town (Mohács)		
				*Z*	*p*		*Z*	*p*
**City** **(Pécs)**	Number of taxa	11.83	8.50	−2.70	0.005	7.83	−2.48	0.010
	Shannon diversity	1.53	0.96	−3.00	0.001	0.84	−2.84	0.003
	Evenness	0.40	0.30	−2.71	0.005	0.26	−2.52	0.010
	Niche breadth	3.39	1.81	−3.00	0.001	1.75	−2.84	0.003
**Town (Mohács)**	Number of taxa	7.83	8.50	0.37	0.792			
	Shannon diversity	0.84	0.96	0.55	0.662			
	Evenness	0.26	0.30	1.10	0.329			
	Niche breadth	1.75	1.81	0.55	0.662			

**Table 4:** The differences between the habitats’ proportion and the relative abundance of small mammals that preferring these habitats in the circle with a radius of 1, 2, and 3 km in each sampling site, with the direction of the differences (dir), which indicates whether the relative abundance of the prey species of Long-eared Owls was over-represented (o-r) or under-represented (u-r) compared to the proportion of their habitats (NS—not significant).

	Settlement Type	Village (Udvar)			Town (Mohács)			City (Pécs)		
		*G*	*p*	dir	*G*	*p*	dir	*G*	*p*	dir
1 km	Urban habitats	4.23	<0.05	u-r	70.41	<0.001	u-r	79.12	<0.001	u-r
	Open habitats	0.94	NS		78.98	<0.001	o-r	30.44	<0.001	o-r
	Forest habitats	21.45	<0.001	o-r	0.19	NS		15.64	<0.001	o-r
	Wetlands	4.77	<0.05	o-r	26.93	<0.001	u-r	3.36	NS	
2 km	Urban habitats	0.02	NS		45.00	<0.001	u-r	61.06	<0.001	u-r
	Open habitats	1.09	NS		15.91	<0.001	o-r	22.62	<0.001	o-r
	Forest habitats	12.17	<0.001	o-r	0.54	NS		4.45	<0.05	o-r
	Wetlands	0.02	NS		11.49	<0.001	u-r	3.36	NS	
3 km	Urban habitats	0.03	NS		24.77	<0.001	u-r	47.93	<0.001	u-r
	Open habitats	0.23	NS		2.91	NS		5.53	<0.05	o-r
	Forest habitats	1.27	NS		2.09	NS		10.99	<0.001	o-r
	Wetlands	0.11	NS		6.35	<0.05	u-r	3.36	NS	

## Data Availability

The original contributions presented in this study are included in the article. Further inquiries can be directed to the corresponding author.
